# Validation of the Scale of Perceived Overqualification (SPOQ) in the Chinese Context

**DOI:** 10.1002/pchj.70031

**Published:** 2025-07-02

**Authors:** Yue Han, Xiongliang Peng, Yan Bai

**Affiliations:** ^1^ School of Labor and Human Resources Renmin University of China Beijing China

**Keywords:** Chinese full‐time employees, factor analysis, perceived overqualification, scale validation, test–retest reliability

## Abstract

The present research aimed to validate the Chinese version of the Scale of Perceived Overqualification (SPOQ), which assesses employees' perceptions of possessing more knowledge, skills, and abilities than their job requires. A preliminary meta‐analytic review highlighted the need to evaluate the SPOQ's psychometric properties in different cultural contexts. In Study 1, exploratory factor analysis supported a single‐factor structure of the SPOQ‐Chinese version, and test–retest analysis confirmed its stability over a 2‐week interval. In Study 2, confirmatory factor analysis demonstrated a good model fit, further verifying the scale's structural validity. Criterion‐related validity was also supported, with significant correlations between perceived overqualification and relevant job‐related and career‐related variables. Overall, our findings suggest the SPOQ‐Chinese version is an appropriate measure of perceived overqualification among Chinese employees.

## 1 Introduction

Overqualification refers to an underemployment condition where one's knowledge, skills, ability (KSA), education, and experience are over job requirements (Erdogan and Bauer 2009; Maynard et al. 2006), and it has recently become a trending phenomenon in the Chinese labor market (Jiang et al. 2022; Li et al. 2022). Overqualification results in several adverse outcomes, affecting not only employees—such as reduced work meaningfulness (Zhang et al. 2023), lower career satisfaction (Erdogan et al. 2018), and diminished life satisfaction (Luksyte et al. 2020)—but also organizations, including increased counterproductive behaviors (Wu and Chi 2020), higher employee turnover rates (Simon et al. 2019), and decreased creative performance (Luksyte and Spitzmueller 2016).

There are two main approaches to measuring overqualification. By definition, overqualification reflects the mismatch between an individual's skills and the job's requirements (Maynard et al. 2006), aligning with the person–job (P‐J) fit framework. The first approach assesses overqualification objectively by comparing an employee's qualifications—such as education, skills, and experience—with the stated requirements of the position, identifying any gaps between the two (Erdogan et al. 2011; Maltarich et al. 2011). Alternatively, some researchers focus on perceived overqualification (POQ), which captures employees' subjective assessment of the gap between their KSAs, experience, education, and the demands of their job (Erdogan and Bauer 2009; Maynard et al. 2006). While both objective and subjective measures aim to capture overqualification, they represent distinct constructs and are only moderately correlated (Erdogan et al. 2011; Arvan et al. 2019; Harari et al. 2017). Maltarich et al. (2011) argue that POQ is a more appropriate measure when examining outcomes related to job attitudes, behaviors, and subjective career success, as it tends to have a stronger connection to employees' well‐being than objective assessments. As for subjective overqualification, Maynard et al. (2006) developed the Scale of Perceived Overqualification (SPOQ), which was one of the most widely used instruments in the field (e.g., Jiang et al. 2022; Li et al. 2022; Luksyte et al. 2020; Wu and Chi 2020). Underemployment was regarded as the conceptual basis of overqualification by most researchers (Feldman 1996), and its contents were all reflected in Maynard et al.'s single‐dimension scale (Maynard et al. 2006). In contrast, the relations among dimensions of multidimensional overqualification scales were not clearly illustrated or justified, and the single‐dimension overqualification scales were always too short or lacked validation (McKee‐Ryan and Harvey 2011).

The widely used Subjective Perceived Overqualification Scale (SPOQ; Maynard et al. 2006) was developed and validated within an individualistic Western context. Since then, it has been applied across various cultural settings (Erdogan et al. 2020; Kim et al. 2021; Luksyte et al. 2020), including several studies conducted in China (e.g., Cheng et al. 2020; Jiang et al. 2022; Liu et al. 2024; Ma, Ganegoda, et al. 2020; Peng et al. 2023). However, despite its frequent use in the Chinese context, no research to date has systematically tested the scale's validity within this cultural setting. Given the SPOQ's origins in a Western, individualistic culture, it is essential to examine whether the scale retains its psychometric integrity when used in an eastern, collectivist culture like China, as cultural differences might affect the psychometric properties of the scale to undermine its validity (van Herk et al. 2004). Within the overqualification literature, recent scholars highlight the importance of cultural dimensions in POQ (Erdogan and Bauer 2021; Liao et al. 2024). While a few studies have touched on how cultural values may shape individuals' experiences of overqualification (e.g., Harari et al. 2017; Yang and Li 2021), the field remains largely “culture blind” (Erdogan and Bauer 2021, 275). Therefore, a critical next step is to assess whether the SPOQ operates similarly across different cultural contexts.

Given above, this paper aims to validate the SPOQ within the Chinese context, thereby providing a reliable instrument for assessing POQ. First, we reviewed the relevant literature and subsequently conducted a meta‐analytic alpha analysis to examine the scale's internal consistency across diverse applications. Second, followed Brislin's (1980) translation and back‐translation process to compile a SPOQ‐Chinese version. Next, exploratory factor analysis (EFA) was performed among Chinese employees to test whether the 9‐item SPOQ‐Chinese version can fall on the same factor structure (Study 1). In addition, we re‐collected data from Study 1 2 weeks later and estimated the test–retest reliability. Finally, to confirm the validation of the SPOQ‐Chinese version, we conducted the confirm factor analysis and estimated validity in Study 2.

## 2 Theoretical Review

Existing empirical studies suggest that national culture is associated with POQ and can influence its relationship with various outcomes, such as individualism (Wu et al. 2015). However, due to cultural differences, there is a lack of cross‐cultural validation of Subjective Perceived Overqualification (SPOQ), raising concerns about its applicability in China for several reasons.

First, influenced by the traditional Confucian values, Chinese employees tend to prioritize harmony, collectivism, and cooperation, while rejecting individualism (King and Bond 1985). Therefore, POQ would be expected to occur less frequently in China (Wu et al. 2015). Nevertheless, numerous empirical studies have found that POQ is not only common among Chinese employees but may even be more prevalent than in Western countries (Cheng et al. 2020; Jiang et al. 2022; Ma, Lin, and Wei 2020; Zhu and Chen 2022; Lin et al. 2017). Second, shaped by the historical legacy of the “iron rice bowl” system in China, many Chinese‐owned organizations have placed limited emphasis on training and instead prioritized tenure (Su and Wright 2012; Warner 2008). As a result, employees may be more likely to perceive themselves as overqualified due to insufficient training opportunities, while organizations often evaluate qualifications based on tenure rather than skill development. Third, influenced by the tradition of imperial examinations, Chinese employees tend to place significant importance on educational attainment as a key indicator of qualification (Gan 2008; Ko 2017). Above reasons challenge the measurement of POQ in Chinese culture, that is, whether the excess of qualifications (KSA, education, and experience) developed based on American culture has the same structure and the same nomological network as POQ in China. If qualifications in Chinese culture express different content or structure, or have different nomological networks, we need to reconsider the cross‐cultural validity of SPOQ, develop new scales or revise existing scales, and re‐verify the research conclusions based on Chinese culture.

Two key theoretical perspectives are central to understanding the nomological network of POQ. Maynard et al. (2006) emphasized the close relation between POQ and P‐J fit, suggesting that employees perceive themselves as overqualified when their skills and qualifications exceed job requirements, leading to lower job satisfaction, reduced commitment, and higher turnover intention. Erdogan and Bauer (2009) further argued that POQ often triggers feelings of relative deprivation, as employees believe their qualifications are undervalued or unrecognized. These two perspectives—P‐J fit theory and relative deprivation theory—form the core of POQ research and are widely applied in the literature (Erdogan et al. 2018; Hu et al. 2015; Kim et al. 2021; Liu et al. 2024; Luksyte et al. 2020; Triana et al. 2017). More recently, a comprehensive review (Erdogan and Bauer 2021) and a refined meta‐analysis (Harari et al. 2017) have further validated the importance of these theories, identifying a range of outcome variables associated with SPOQ, such as career satisfaction, turnover intention, organizational commitment, affectivity, and perceptions of justice. These studies offer valuable theoretical guidance for establishing the nomological validity of SPOQ.

Before proceeding with the validation of the SPOQ, we conducted a meta‐analytic review of its internal consistency across existing studies (Greco et al. 2018). The goal was to evaluate the reliability of the scale—specifically Cronbach's alpha—and to assess the stability and generalizability of its measurement across high‐quality, peer‐reviewed research.

## 3 Meta‐Analytic Review of the Scale's Reliability

### 3.1 Literature Search and Inclusion Criteria

We took several steps to systematically identify eligible studies and used search terms relevant to our focal variable, POQ. These search terms included “perceived overqualification,” “overqualification,” “overqualified,” “underemployment,” we observed 421 articles at this step. Second, we searched for articles published in top‐tier journals of management and psychology, such as *Academy of Management Journal*, *Journal of Applied Psychology*, *Administrative Science Quarterly*, *Journal of Management*, *Personnel Psychology*, *Journal of Personality and Social Psychology*, *Organization Science*, *Journal of Organizational Behavior*, *Human Resource Management*, *Human Resource Management Journal*, *Journal of Vocational Behavior*, *Journal of Occupational and Organizational Psychology*; then we observed 78 articles. Third, focusing on our research, we included articles using the SPOQ we intend to validate (Maynard et al. 2006), we identified and reviewed 27 articles published in top‐tier journals that employed the SPOQ (Maynard et al. 2006); see the details of articles in Table A1.

### 3.2 Meta‐Analytic Results for Alpha

As shown in Table 1, 11.11% of the studies reported alpha values below 0.80, and all these are Chinese samples. Among the studies that reported alpha values below 0.90, 78.6% are from Chinese samples. Among the 27 articles reviewed, 51% used data from Chinese participants, with the weighted average alpha of 0.83. In contrast, 37% of the studies were conducted in individualistic Western countries—such as the United States, the Netherlands, Spain, and Germany (Minkov et al. 2017)—and reported a higher weighted average alpha of 0.91. According to Nunnally (1978), a reliability coefficient of 0.80 is generally acceptable for basic research, whereas applied contexts involving critical decisions require a higher threshold of at least 0.90. In light of this benchmark, the observed differences in Cronbach's alpha across studies preliminarily suggest some variability in the internal consistency of the overqualification scale across cultural contexts (Beretvas et al. 2002; Caruso et al. 2001).

**TABLE 1:** Meta‐analytic results for alpha across studies.

SPOQ	*k*	*N*	*α*	95% CI	80% CV	< 0.80%	< 0.90%
Total	27	9394	0.87	[0.85, 0.89]	[0.79, 0.94]	11.1%	55.6%
Chinese context	14	4037	0.83	[0.80, 0.86]	[0.75, 0.91]	21.4%	78.6%
Western context	10	3490	0.91	[0.89, 0.93]	[0.87, 0.95]	0.0%	30.0%

*Note*: k, number of reliabilities; *N*, total sample size; *α*, weighted mean alpha; 95% CI, 95% confidence interval of *α*; 80% CV, 80% credibility interval of *α*; % below represents the proportion of studies below 0.80 and 0.90 cut‐offs; three studies were not included as the sample we could not categorize in either individualistic Western or Chinese.

Overall, reported Cronbach's alpha estimates range from 0.71 to 0.94, with weighted average alpha of 0.87 (95% CI [0.85, 0.89]), show very wide credibility intervals (95% CV [0.79, 0.94]). Results show very wide credibility intervals among Chinese samples (95% CV [0.75, 0.91]) but relatively narrow credibility intervals among samples in western context (95% CV [0.87, 0.95]), indicating that the internal consistency of the SPOQ is not entirely stable across different research contexts, which may be attributable to differences in sample characteristics, cultural and linguistic adaptations. Taken together, these findings highlight the need for further psychometric evaluation of the scale in culturally distinct settings.

## 4 Study 1: Translation, EFA, and Test–Retest Reliability

### 4.1 Method

#### 4.1.1 Measure and Procedure

To guarantee the equivalence of translated scale, we followed Brislin's (1980) translation and back‐translation process. First, the first author translated the English scale into Chinese, and then the third author translated the Chinese scale back into English for comparison with the original scale. After that, the inconsistent parts were discussed and modified, and this process was iterated until they were in agreement. Finally, the second author with a management doctoral degree compared the final Chinese scale and the original English scale to confirm that they expressed the same meaning.

Studies 1 and 2 used different participant samples, both recruited through Credamo, a Chinese crowdsourcing platform comparable to Amazon Mechanical Turk in functionality. The platform enables researchers to screen for low‐quality responses and reject up to 20% of invalid submissions. Participants whose responses were identified as invalid did not receive compensation, and their data were excluded from analysis. After applying these quality control procedures, we retained a final valid sample of 400 participants in each study. All our participants were adults and full‐time employees, and they were assured of their anonymity and the confidentiality of their responses. To encourage participation and ensure a high response rate, each participant was offered a 10 RMB participation fee upon successful completion of the survey each time. Written informed consent was obtained from the participants. Both of the studies were conducted in accordance with the Declaration of Helsinki, and approved by the Ethics Committee of School of Labor and Human Resources, Renmin University of China.

To avoid the bots and insufficient effort responding, we used a response pattern approach which asks respondents to select specific options (Huang et al. 2012). Crowdsourcing data had not only similar data quality to specific‐organization pools but also much more external validation (Behrend et al. 2011). For Study 1, on May 13, 2022, we launched a questionnaire on the crowdsourcing platform and collected 400 full‐time Chinese employees who answered the questionnaire effectively. Two weeks later, we sent the questionnaire again to these 400 respondents, 351 of whom finished the answers twice.

##### 4.1.1.1 POQ

The SPOQ was used to measure subjective perceptions of overqualification. It contained nine items rating on a 7‐point Likert scale ranging from *strongly disagree* to *strongly agree*. SPOQ was a single‐factor scale that assessed employees' perception of their surplus KSA, education, and experience in three items apart. The sample items were “I have more abilities than I need in order to do my job,” “My job requires less education than I have,” and “The work experience that I have is not necessary to be successful on this job.”

#### 4.1.2 Participants

Among 400 employees, 234 (58.5%) were female, and 166 (41.5%) were male. Most participants had a bachelor's degree or above (*N* = 355, 88.8%). Most of the participants were between the ages of 21–30 (*N* = 207, 51.7%) and 31–40 (*N* = 168, 42.0%). The average length of work experience was 7.3 years.

### 4.2 Results

#### 4.2.1 EFA

The Kaiser–Meyer–Olkin (KMO) measure of sample adequacy was 0.88, and Bartlett's test of sphericity was significant (*p* < 0.001), indicating that the SPOQ‐Chinese version was suitable for factor analysis. We conducted EFA using the principal component analysis (PCA) and direct Oblimin rotations in SPSS 26.0. Table 2 revealed the final factor loadings for each item, clustered on one factor. According to Beavers et al. (2013) and Hinkin (1998), there was no item with cross‐loadings or loadings less than 0.40 that needed to be deleted. All the factor loadings were above 0.70 and on the only factor. The single‐factor structure accounted for 58.48% of the total variance.

**TABLE 2:** Results of exploratory factor analyses of Study 1.

Items and their Chinese translation	Loading
1. My job requires less education than I have 1. 我的工作对学历的要求低于我拥有的学历	0.79
2. The work experience that I have is not necessary to be successful on this job 2. 做好当前这份工作并不必然需要我之前的工作经验	0.78
3. I have job skills that are not required for this job 3. 我有一些当前这份工作用不到的工作技能	0.74
4. Someone with less education than myself could perform well on my job 4. 比我学历低的人也能做好我的这份工作	0.72
5. My previous training is not being fully utilized on this job 5. 我之前的训练在当前这份工作上没有完全用上	0.76
6. I have a lot of knowledge that I do not need in order to do my job 6. 我的很多知识在当前的工作中用不到	0.82
7. Someone with less work experience than myself could do my job just as well 7. 比我工作经验少的人也能做好我的这份工作	0.76
8. My education level is above the education level required by my job 8. 我的学历水平超过了当前工作需要的学历水平	0.80
9. I have more abilities than I need in order to do my job 9. 我的能力超过了当前工作所需要的能力	0.71
Eigenvalues	5.26
% Explained variance	58.48

*Note: n* = 400. Extraction method: Principal component analysis; Rotation method: Direct Oblimin rotations.

#### 4.2.2 Test–Retest Reliability

The Cronbach alpha of SPOQ‐Chinese version was 0.91 in Time 1 (*n* = 400) and 0.92 in Time 2 (*n* = 351). We matched the two waves of data and conducted a correlation analysis based on the 351 samples. Test–retest reliability estimated for calculating the SPOQ‐Chinese version correlation at different times was 0.83 (the interitem correlations between different times ranged from 0.61 to 0.77, and the average correlation was 0.70).

## 5 Study 2: Confirmatory Factor Analysis and Validity Estimation

### 5.1 Method

#### 5.1.1 Participants

Study 2 was conducted on June 13, 2022, with an independent sample of 400 full‐time Chinese employees, also recruited via the same crowdsourcing platform. Among 400 employees, 261 (65.3%) were female, and 139 (34.8%) were male. The participants were 32.03 years old on average. Most participants had a bachelor's degree or above (*n* = 355, 88.8%). The participants had an average work experience of 8.22 years.

#### 5.1.2 Procedure and Measures

First, we conducted a confirmatory factor analysis (CFA) to confirm the factor structure of the SPOQ‐Chinese version. Parceling is a common data processing method used in CFA (e.g., Gupta et al. 2015; Kim et al. 2019). It has the advantage of item discrimination and nonnormal distribution data (Little et al. 2002). To avoid redefining the nature of POQ, we used shared uniqueness strategy to parcel (Hall et al. 1999). Thus, we packaged the nine‐item SPOQ‐Chinese version into three parcels: three‐item for KSA, three‐item for education, and three‐item for work experience. Moreover, to estimate criteria‐related validity and provide further validity evidence, drawing upon critical reviews and empirical studies (Andel et al. 2021; Erdogan and Bauer 2021; Harari et al. 2017; Maynard et al. 2006), we identified the following criteria: job satisfaction, career satisfaction, relative deprivation, job boredom, distributive justice perception, P‐J fit, affective commitment, turnover intention, organization‐based self‐esteem, anger toward the organization, positive affectivity, and negative affectivity. The process of the scale translation and quality control were the same as Study 1.

##### 5.1.2.1 POQ

The SPOQ developed by Maynard et al. (2006) was a nine‐item scale same as Study 1. Interitem correlations ranged from 0.32 (Item 4 and Item 9) to 0.74 (Item 1 and Item 8). The Cronbach alpha of Maynard et al. (2006) SPOQ‐Chinese version was 0.89. Besides, we also tested the SPOQ developed by Johnson and Johnson (1996). It contained two dimensions which were named “mismatch” and “non‐growth.” The sample items were “My work experience is more than necessary to do my present job” and “Continuing education related to my job has improved my job performance.” However, the Cronbach alpha of Johnson and Johnson's (1996) SPOQ was 0.47—lower than the 0.60 cut‐off. Thus, it did not adopt in the following analysis.

##### 5.1.2.2 Job Satisfaction

The job satisfaction scale was a five‐item measure developed by Judge et al. (1998) to assess the degree to which individual was satisfied with their job. A sample item is “I find real enjoyment in my work.” Respondents rated the items on a 7‐point scale (1 = *strongly disagree*; 7 = *strongly agree*). The Cronbach alpha was 0.84.

##### 5.1.2.3 Career Satisfaction

The career satisfaction scale was a five‐item measure developed by Greenhaus et al. (1990) to assess the degree to which individual was satisfied with their career. A sample item is “I am satisfied with the progress I have made toward meeting my overall career goals.” Respondents rated the items on a 7‐point scale (1 = *strongly disagree*; 7 = *strongly agree*). The Cronbach alpha was 0.74.

##### 5.1.2.4 Relative Deprivation

The relative deprivation scale was a three‐item measure developed by Feldman and Turnley (2004) to assess the degree to which individuals' sense of deprivation is relative to others. A sample item is “Generally speaking, I want a better job situation than the one I have now.” Respondents rated the items on a 7‐point scale (1 = *strongly disagree*; 7 = *strongly agree*). The Cronbach alpha was 0.81.

##### 5.1.2.5 Job Boredom

The job boredom scale was a six‐item measure developed by Reijseger et al. (2013) to assess the degree of job boredom perceived by individuals. A sample item is “At work, time goes by very slowly.” Respondents rated the items on a 7‐point scale (1 = *strongly disagree*; 7 = *strongly agree*). The Cronbach alpha was 0.82.

##### 5.1.2.6 Distributive Justice Perception

The distributive justice perception scale was a five‐item measure developed by Niehoff and Moorman (1993) to assess the degree to which individuals' perception of justice is distributed. A sample item is “I think that my level of pay is fair.” Respondents rated the items on a 7‐point scale (1 = *strongly disagree*; 7 = *strongly agree*). The Cronbach alpha was 0.90.

##### 5.1.2.7 P‐J Fit

The P‐J fit scale was a four‐item measure developed by Saks and Ashforth (2002) to assess the degree of fitness between the individual and the job. A sample item is “To what extent is the job a good match for you?” Respondents rated the items on a 7‐point scale (1 = *to a very little extent*; 7 = *to a very large extent*). The Cronbach alpha was 0.75.

##### 5.1.2.8 Affective Commitment

The affective commitment scale was a six‐item measure developed by Meyer et al. (1993) to assess the degree to which an individual commits to the organization in the aspect of affect. A sample item is “I would be very happy to spend the rest of my career with this organization.” Respondents rated the items on a 7‐point scale (1 = *strongly disagree*; 7 = *strongly agree*). The Cronbach alpha was 0.86.

##### 5.1.2.9 Turnover Intention

The turnover intention scale was a three‐item measure developed by Chen et al. (1998) to assess the degree to which the individual intention to leave out the organization. A sample item is “It is very possible that I will look for a new job next year.” Respondents rated the items on a 7‐point scale (1 = *strongly disagree*; 7 = *strongly agree*). The Cronbach alpha was 0.86.

##### 5.1.2.10 Organization‐Based Self‐Esteem

The organization‐based self‐esteem scale was a 10‐item measure developed by Pierce et al. (1989) to assess the degree to which individual self‐esteem is framed in the organization context. A sample item is “I count around here.” Respondents rated the items on a 7‐point scale (1 = *strongly disagree*; 7 = *strongly agree*). The Cronbach alpha was 0.91.

##### 5.1.2.11 Anger Toward the Organization

The anger toward the organization scale was a three‐item measure developed by Mitchell et al. (2015) to assess the degree to which individual anger emotion toward its organization. A sample item is “It is very possible that I will look for a new job next year.” Respondents rated the items on a 7‐point scale (1 = *strongly disagree*; 7 = *strongly agree*). The Cronbach alpha was 0.77.

##### 5.1.2.12 Positive Affectivity and Negative Affectivity

The positive affectivity and negative affectivity scale was a 10‐item measure developed by Thompson (2007) to assess the degree to which the individual's The sample item of positive affectivity is “Active,” and the sample item of negative affectivity is “Afraid.” Respondents rated the items on a 7‐point scale (1 = *never*; 7 = *always*) to evaluate how often they had mentioned affectivity in the last 2 weeks. The Cronbach alpha of positive affectivity and negative affectivity was 0.87 and 0.76, respectively.

### 5.2 Results

#### 5.2.1 CFA

To optimize measurement structure and proceed with CFA, the nine‐item SPOQ‐Chinese version was randomly parceled into three groups, which was followed by Little et al.'s (2002) recommendation. We conducted CFA using maximum likelihood estimation by Mplus 7.4. To reduce the parameters and estimate the CFA available, we set the variance of the latent POQ factor to be 1. The indices supported that one‐factor model had a good fit, *χ* ^2^(1, *N* = 400) = 3.037, *p* = 0.081, root mean square error of approximation = 0.071; comparative fit index = 0.997; Tucker–Lewis index = 0.992. Standardized factor loading of three parcel was 0.85, 0.94, 0.76, the average variance extracted (AVE) = 0.73, and the composite reliability (CR) = 0.89, demonstrating good convergent validity and internal consistency. To assess potential common method bias, we conducted Harman's single‐factor test (Podsakoff et al. 2012). The analysis showed that a single factor accounted for 36.06% of the total variance, suggesting that common method bias was not a serious concern in this study.

#### 5.2.2 Validity Estimates

As illustrated in Table 3, the result indicated that POQ has significantly correlated with job satisfaction (*r* = −0.23, *p* < 0.01), career satisfaction (*r* = −0.19, *p* < 0.01), relative deprivation (*r* = 0.36, *p* < 0.01), job boredom (*r* = 0.27, *p* < 0.01), distributive justice perception (*r* = −0.24, *p* < 0.01), P‐J fit (*r* = −0.25, *p* < 0.01), affective commitment (*r* = −0.23, *p* < 0.01), turnover intention (*r* = 0.25, *p* < 0.01), organization‐based self‐esteem (*r* = 0.18, *p* < 0.01), anger toward the organization (*r* = 0.19, *p* < 0.01), positive affectivity (*r* = −0.20, *p* < 0.01), and negative affectivity (*r* = 0.11, *p* < 0.05). All nomological and perception‐related variables would be significantly and moderately correlated with POQ, indicating that results of concurrent validity and convergence validity tests agreed with the expectations. In order to test discriminant validity, we compared the square root of POQ's AVE with the correlations with other constructs. When the AVE of the targeted construct exceeds the correlation coefficient between the target construct and related construct, the discriminant validity between the target construct and related construct is proved (Kline 2005). The square root of AVE for POQ (0.85) exceeds the intercorrelations of POQ with other constructs. To further assess the discriminant validity of the SPOQ‐Chinese version, we conducted an unsupervised *k*‐means clustering analysis at the item level. The use of clustering techniques in psychometric validation has been demonstrated in recent work (e.g., Jang et al. 2024), particularly to visualize and quantify the separation between constructs beyond traditional CFA‐based criteria. Results demonstrated strong item‐level cohesion for SPOQ, with 100% clustering consistency observed in most comparisons and silhouette scores ranging from 0.275 to 0.383. A few comparisons (e.g., with P‐J fit, job boredom) revealed slightly lower separation (detailed information and full clustering visualizations based on PCA are provided in the Supporting Information), this may be attributed to cultural nuances in how occupational qualities and job stress are perceived in the Chinese context. Generally, the clustering results—complemented by traditional validation methods—support the discriminant validity of the SPOQ in Chinese populations.

**TABLE 3:** Correlations between perceived overqualification and other variables of study 2.

Variable	Mean	SD	1	2	3	4	5	6	7	8	9	10	11	12	13
1. POQ	3.84	1.18	(0.89)												
2. JS	5.77	0.92	−0.23**	(0.84)											
3. CS	5.33	0.90	−0.19**	0.66**	(0.74)										
4. RD	2.97	1.32	0.36**	−0.68**	−0.54**	(0.81)									
5. JB	2.56	1.00	0.27**	0.77**	−0.58**	0.70**	(0.81)								
6. DJP	5.51	1.02	−0.24**	0.68**	0.65**	−0.66**	−0.65**	(0.90)							
7. PJF	5.71	0.72	−0.25**	0.79**	0.64**	−0.66**	−0.69**	0.68**	(0.75)						
8. AC	5.51	1.07	−0.23**	0.79**	0.58**	−0.71**	−0.72**	0.60**	0.73**	(0.86)					
9. TI	2.38	1.17	0.25**	−0.78**	−0.60**	0.75**	0.72**	−0.65**	−0.78**	−0.83**	(0.86)				
10. OS	5.89	0.73	0.18**	−0.34**	−0.20**	0.26**	0.40**	−0.21**	−0.32**	−0.28**	0.28**	(0.91)			
11. ATO	1.91	0.84	0.19**	−0.69**	−0.43**	0.53**	0.60**	−0.53**	−0.60**	−0.68**	0.67**	0.33**	(0.77)		
12. PA	5.61	0.95	−0.20**	0.76**	0.54**	−0.63**	−0.70**	0.57**	0.73**	0.75**	−0.73**	−0.28**	−0.56**	(0.87)	
13. NA	1.96	0.67	0.11*	−0.49**	−0.41**	0.38**	0.49**	−0.54**	−0.48**	−0.40**	0.45**	0.17**	0.48**	−0.43**	(0.76)

*Note: N* = 400. Cronbach alpha is reported in parentheses on the diagonal.

Abbreviations: AC, affective commitment; ATO, anger toward the organization; CS, career satisfaction; DJP, disruptive justice perception; JS, job satisfaction; JB, job boredom; NA, negative affectivity; OS, organization‐based self‐esteem; PA, positive affectivity; PJF, P‐J fit; POQ, Perceived overqualification; RD, relative deprivation; SD, standard deviation; TI, turnover intention.

* *p* < 0.05.

** *p* < 0.01.

Overall, the results showed that the SPOQ‐Chinese version was validated in Chinese samples.

## 6 Discussion

A growing body of research highlights the increasing relevance of POQ, often viewed as a form of subjective underemployment (Lin et al. 2017). POQ has emerged as a key factor influencing employees' career outcomes and well‐being (Erdogan et al. 2018; Erdogan and Bauer 2021). While global data show that about 47% of employees feel overqualified, this figure rises sharply to 84% among Chinese employees (Randstad Workmonitor 2012), reflecting widespread concern in China. There have been several articles POQ in a Chinese context (e.g., Cheng et al. 2020; Jiang et al. 2022; Liu et al. 2024; Ma, Ganegoda, et al. 2020; Peng et al. 2023). Nevertheless, there remains limited evidence regarding the cross‐cultural validity of the SPOQ. To address this, we conducted a meta‐analytic review of the SPOQ's internal consistency across existing studies (Greco et al. 2018). The analysis revealed broad credibility intervals among Chinese samples, in contrast to narrower intervals found in Western contexts, suggesting that the SPOQ's internal consistency may not be fully stable across cultural settings. These findings underscore the need for further psychometric evaluation of the SPOQ in diverse cultural contexts. In response, our study translated and validated the SPOQ in a Chinese context using a four‐step process. First, we translated the original SPOQ into Chinese following Brislin's (1980) back‐translation method, providing a culturally adapted measurement tool. Second, we conducted an EFA in Study 1 to examine the scale's factor structure and assessed its test–retest reliability. Third, in Study 2, we used CFA to further validate the factor structure. Finally, we examined criterion‐related, convergent, and discriminant validity to evaluate the overall measurement quality of the SPOQ‐Chinese version.

Our findings showed that POQ correlated significantly with a broad set of theoretically relevant variables. Consistent with previous meta‐analytic findings (Erdogan and Bauer 2021), POQ was negatively associated with job attitudes and career success, and positively associated with turnover intentions. Specifically, we found significant correlations between POQ and distributive justice perceptions, career satisfaction, and turnover intention. These results aligned closely with the outcomes reported in Harari et al.'s (2017) meta‐analysis, thereby strengthening the credibility of our findings. We also explored the emotional responses associated with POQ, drawing on the work of Andel et al. (2021). Our results confirmed significant associations between POQ and job boredom, organization‐based self‐esteem, and feelings of anger toward the organization, further supporting the scale's construct validity. In addition, we tested two core theoretical frameworks frequently used in POQ research: P‐J fit and relative deprivation (Maynard et al. 2006). The significant correlations between POQ and both constructs support their central roles in understanding POQ. By including 10 distinct validation criteria, our design helped mitigate the risk of inflated associations due to conceptual overlap, thereby strengthening the credibility of our validity evidence. Furthermore, measurement invariance testing showed no significant differences in model fit across gender, age, education level and years of work experience, suggesting that respondents interpret and respond to the SPOQ items consistently. This indicates that the SPOQ‐Chinese version demonstrates structural stability and provides equally valid measurements across key demographic categories.

### 6.1 Theoretical Contribution

First, we conducted a meta‐analytic review of the SPOQ's internal consistency across published studies (Greco et al. 2018). The results showed wide credibility intervals among Chinese samples, compared to narrower intervals in Western contexts. This suggests that the SPOQ's internal consistency may vary across cultural settings as cultural characteristics were seen as important contingencies in POQ research (Erdogan and Bauer 2021; Liao et al. 2024).

Second, we developed and validated a Chinese version of the SPOQ, offering researchers a culturally adapted tool for assessing POQ in China. This addresses an important measurement gap, as China combines a highly educated workforce with a large number of low‐skill jobs (Xu et al. 2018), making POQ a particularly relevant issue (Li et al. 2019, 2020). Relying on an SPOQ version developed for U.S. samples may lead to issues in validity—such as mismatched factor structures—potentially resulting in inconsistent measurement and flawed inferences (Hui and Triandis 1985). Our validated SPOQ‐Chinese version helps address this gap, providing a reliable tool for future research in the Chinese context.

Third, this work also contributes to the broader underemployment literature by extending the cross‐cultural applicability of POQ measurement. In line with Buyukgoze‐Kavas et al.'s (2021) validation of SPOQ in Turkey, our findings support the idea that POQ is a unidimensional construct comprising interrelated human capital elements—such as education, experience, and skills. Despite cultural differences between China and the United States (Hofstede 1984), our analysis confirmed a consistent factor structure across both contexts. This suggests that POQ, as measured by the SPOQ, may have stable psychometric properties across cultures, advancing the cross‐national study of overqualification and underemployment.

### 6.2 Practical Contribution

This study presents the SPOQ‐Chinese version as a well‐validated tool for the first time, offering a foundation for a deeper understanding of POQ in the Chinese context. Beyond academic research, this contribution holds practical value for organizational managers, policymakers, and career counselors. These people can use the SPOQ‐Chinese version to assess POQ among Chinese employees more accurately. Such assessments can guide interventions to help individuals better align their roles, career paths, or psychological expectations—ultimately improving job satisfaction and overall career well‐being.

### 6.3 Limitation

This research has several limitations. First, most participants held bachelor's degrees, leading to limited variability in educational background. This sampling bias may be due to the nature of online crowdsourcing platforms, which tend to attract more highly educated users (Tahaei and Vaniea 2022). In addition, the absence of detailed occupational data, such as job type and industry, constrained our ability to examine potential differences across employment sectors. Future research would benefit from including more diverse samples in terms of education and occupational backgrounds to enhance the generalizability of findings. Second, while the study assessed correlations between POQ and various attitudinal and emotional variables to examine construct and criterion‐related validity, we were unable to include objective behavioral indicators (e.g., turnover or job performance). Incorporating such measures in future research could provide a more comprehensive assessment of predictive validity. Third, we conducted a preliminary meta‐analysis to assess the internal consistency of the SPOQ across existing studies (Greco et al. 2018) and examined its factor structure within a Chinese context, drawing comparisons with findings from Western samples (e.g., Maynard et al. 2006). However, due to data limitations, we were unable to formally test cross‐cultural measurement invariance. Future research should consider using multi‐group CFA to more thoroughly evaluate the SPOQ's applicability across different cultural settings.

## 7 Conclusion

This research offers a validated Chinese version of the SPOQ, addressing a critical gap in the measurement of POQ within the Chinese cultural context. Our results demonstrate that the SPOQ‐Chinese version has strong psychometric properties, including reliable factor structure, internal consistency, and construct validity. By conducting a meta‐analytic review of SPOQ's internal consistency and empirically validating the Chinese version through two studies, we provide a culturally appropriate tool for assessing POQ among Chinese employees. These findings not only support the scale's application in China but also contribute to broader cross‐cultural research on overqualification.

## Conflicts of Interest

The authors declare no conflicts of interest.

## Supporting information


**Data S1.** Supporting Information.

## Appendix A

**TABLE A1:** Summary of coefficient alpha of SPOQ (Maynard et al. 2006).

Author(s)	Title	Journal	Year	Source	Sample description	Size	*α*
Arvan et al.	Too good for your job? Disentangling the relationships between objective overqualification, perceived overqualification, and job dissatisfaction	*Journal of Vocational Behavior*	2019	the United States	Full‐time employed (30 or more hours per week) recent college graduates with a bachelor's degree in psychology from a large public university	162	0.91
Wiegan	When overqualification turns dark: A moderated‐mediation model of perceived overqualification, narcissism, frustration, and counterproductive work behavior	*Personality and Individual Differences*	2023	the United States	US adults originating on Amazon's MTurk crowdsourcing platform	696	0.93
Liu et al.	Overqualification and counterproductive work behaviors: Examining a moderated mediation model	*Journal of Organizational Behavior*	2015	China	Participants from the research and development (R&D) department of a large manufacturing company located in Harbin, China	224	0.75
Erdogan et al.	Employee overqualification and manager job insecurity: Implications for employee career outcomes	*Human Resource Management*	2020	Turkey	Employees and managers working for a holding company established to support a private university, as well as from the administrative personnel of the university in Ankara, Turkey	594	0.9
Luksyte et al.	Perceived overqualification and collectivism orientation: Implications for work and nonwork outcomes	*Journal of Management*	2022	Australia, Germany, Lithuania, etc.	Participants from 95 universities across eight countries (i.e., Australia, Germany, Lithuania, Switzerland, Taiwan, Turkey, United Kingdom, and United States)	852	0.9
Zhang et al.	Perceived overqualification, counterproductive work behaviors and withdrawal: a moderated mediation model	*Journal of Managerial Psychology*	2024	China	Full‐time employees recruiting through WeChat	176	0.82
Cheng et al.	Perceived overqualification and cyberloafing: A moderated‐mediation model based on equity theory	*Journal of Business Ethics*	2020	China	Data from the Anhui province of China	318	0.75
Jiang et al.	Leaders' response to employee overqualification: An explanation of the curvilinear moderated relationship	*Journal of Occupational and Organizational Psychology*	2022	China	Participants from the Credamo platform, a market research service company in China	372	0.81
Chen et al.	Why and when perceived overqualification drives positive relational outcomes: An optimal distinctiveness perspective	*Journal of Business Research*	2025	China	Supervisor‐subordinate dyads from a large Chinese company	325	0.9
Peng et al.	Perceived overqualification and proactive behavior: The role of anger and job complexity	*Journal of Vocational Behavior*	2023	China	Data from a large state‐owned company specializes in sea transportation and salvage and has many small teams	473	0.82
Erdogan et al.	Perceived overqualification at work: implications for extra‐role behaviors and advice network centrality	*Journal of Management*	2020	Turkey	Employees and supervisors working in one of the 39 district municipalities in Istanbul, Turkey	421	0.82
Jahantab et al.	Are we friends? Relative overqualification, citizenship, and the mediating role of friendship network centrality	*Journal of Business and Psychology*	2024	the United States	Data from 39 restaurants located in the Southwestern US	189	0.81
Ma et al.	To stand out or fit in? How perceived overqualification motivates proactive and affiliative performance	*Human Resource Management*	2023	China	Employees from six large manufacturing companies in southern China	249	0.91
Erdogan et al.	Perceived overqualification, relative deprivation, and person centric outcomes: The moderating role of career centrality	*Journal of Vocational Behavior*	2018	Spain	Undergraduate and master students who found jobs after graduation	143	0.94
Maynard et al.	Perceived overqualification and withdrawal behaviours: Examining the roles of job attitudes and work values	*Journal of Occupational and Organizational Psychology*	2013	the United States	Alumni of a small public university in the north‐eastern United States	368	0.93
Deng et al.	A relational model of perceived overqualification: The moderating role of interpersonal influence on social acceptance	*Journal of Management*	2018	China	Engineers who worked collaboratively in teams to set up and maintain IT systems for corporate clients	194	0.87
Simon et al.	Built to last: Interactive effects of perceived overqualification and proactive personality on new employee adjustment	*Personnel Psychology*	2019	the United States	New hires who were scheduled to attend a new employee orientation at a large financial institution based in the United States	622	0.87
Ma et al.	Linking perceived overqualification with task performance and proactivity? An examination from self‐concept‐based perspective	*Journal of Business Research*	2020	China	Participants from three companies in technology‐intensive industries in China	351	0.71
Jiang et al.	Challengers, not followers? The effect of leaders' perceptions of team overqualification on leaders' empowering behavior	*Journal of Managerial Psychology*	2024	China	Team leaders or who had experience as leaders using Credamo	95	0.82
Andel et al.	Bored, angry, and overqualified? The high‐ and low‐intensity pathways linking perceived overqualification to behavioural outcomes	*European Journal of Work and Organizational Psychology*	2022	the United States	Full‐time administrative employees at a large southeastern public university	558	0.92
Gkorezis et al.	Implications of perceived overqualification for employee's close social ties: The moderating role of external organizational prestige	*Journal of Vocational Behavior*	2019	Greece	Employees in a Greek organization in the gambling industry	116	0.92
Liu et al.	Perceived overqualification and employee outcomes: The dual pathways and the moderating effects of dual‐focused transformational leadership	*Human Resource Management*	2024	China	MBA alumni of a business school located in central China	469	0.9
Li et al.	I despise but also envy you: A dyadic investigation of perceived overqualification, perceived relative qualification, and knowledge hiding	*Personnel Psychology*	2022	China	Service representatives who work in a functional team setting, wherein all members are in the same position/rank and carry out similar tasks, from a Chinese biotechnology company	160	0.86
Alfes et al.	Reducing perceptions of overqualification and its impact on job satisfaction: the dual roles of interpersonal relationships at work	*Human Resource Management Journal*	2016	Netherlands	Employees working at two organisations in different industries located in the Netherlands	183	0.88
Ma et al.	Speaking for organization or self? Investigating the effects of perceived overqualification on pro‐organizational and self‐interested voice	*Journal of Business Research*	2023	China	Employees and their immediate supervisors from 11 high‐tech companies in Northern China	411	0.85
Debus et al.	Reacting to perceived overqualification: Uniting strain‐based and self‐regulatory adjustment reactions and the moderating role of formal work arrangements	*Journal of Business and Psychology*	2023	Germany	Participants from a market research company	453	0.91
Ma et al.	Effects of perceived overqualification on career distress and career planning: Mediating role of career identity and moderating role of leader humility	*Human Resource Management*	2020	China	Participants from a large company in a southern province of China	220	0.81
