# Supplementary material for: Validation of the Scale of Perceived Overqualification (SPOQ) in the Chinese Context
Source: Psych J. 2025 Jul 2;14(5):813–25. doi: 10.1002/pchj.70031 (PMC12520846; doi:10.1002/pchj.70031)
Supplement: Supplementary file 1 — Data S1. Supporting Information. [file PCHJ-14-813-s001.docx]

PCA Visualizations of Item-Level K-means Clustering for Discriminant Validity


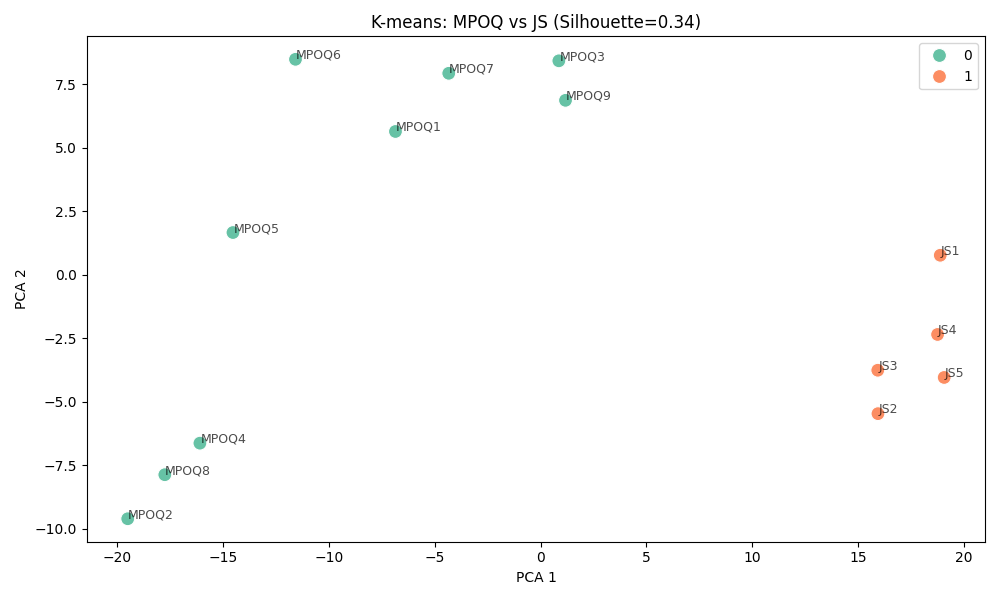


Figure 1. POQ vs Job Satisfaction


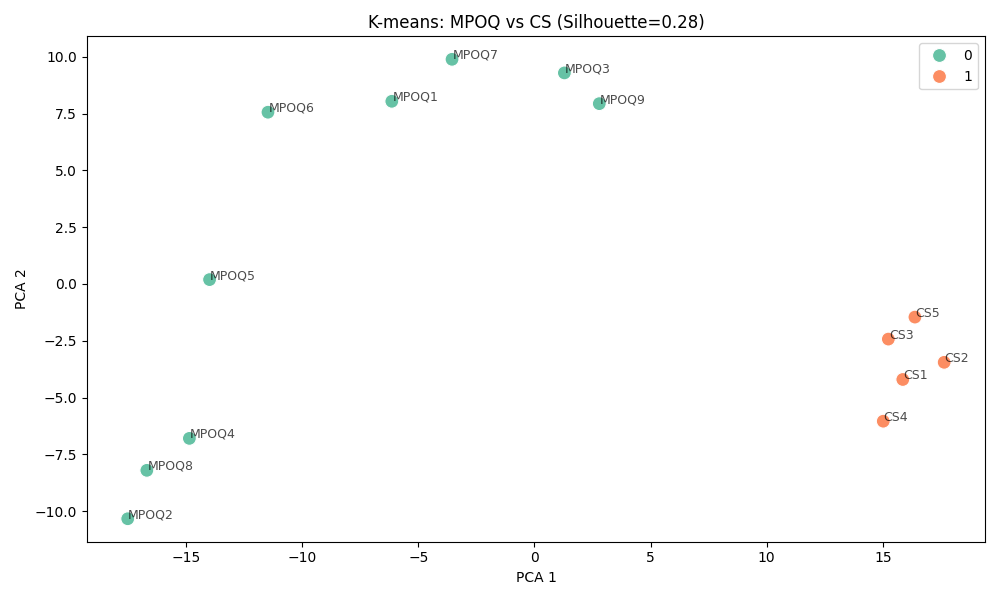


Figure 2. POQ vs Career Satisfaction


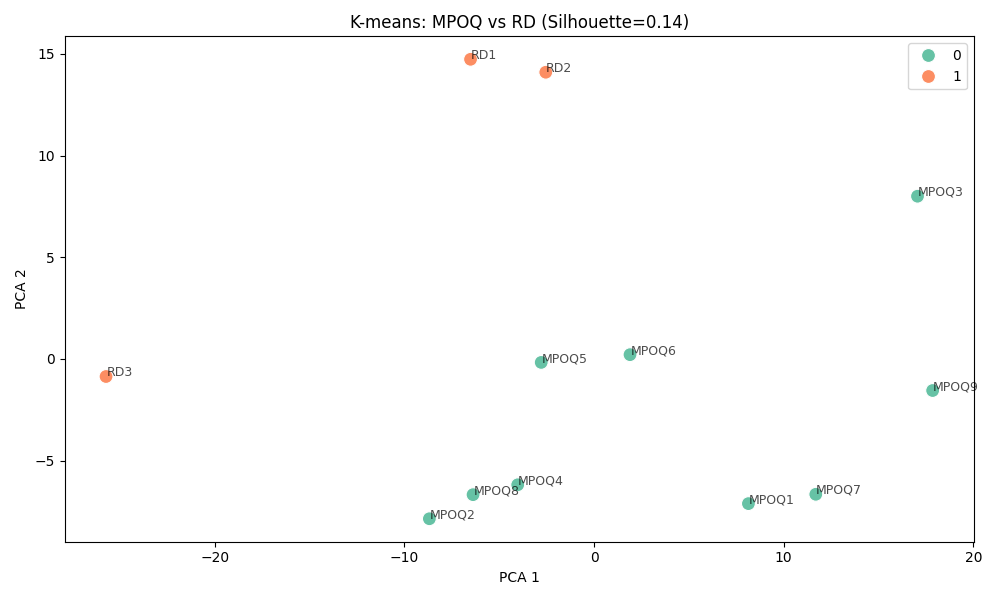


Figure 3. POQ vs Relative Deprivation


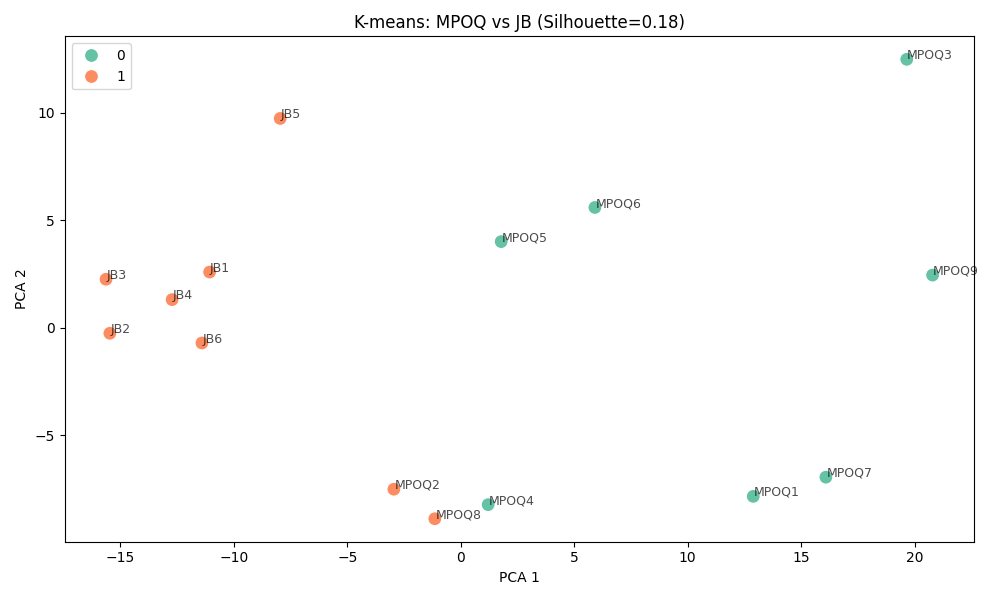


Figure 4. POQ vs Job Boredom


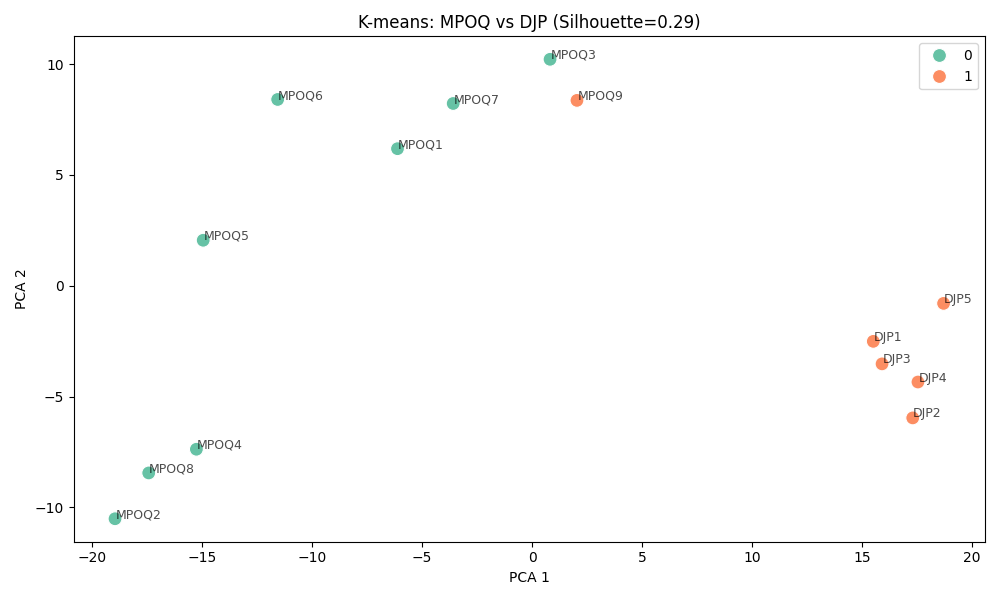


Figure 5. POQ vs Disruptive Justice Perception


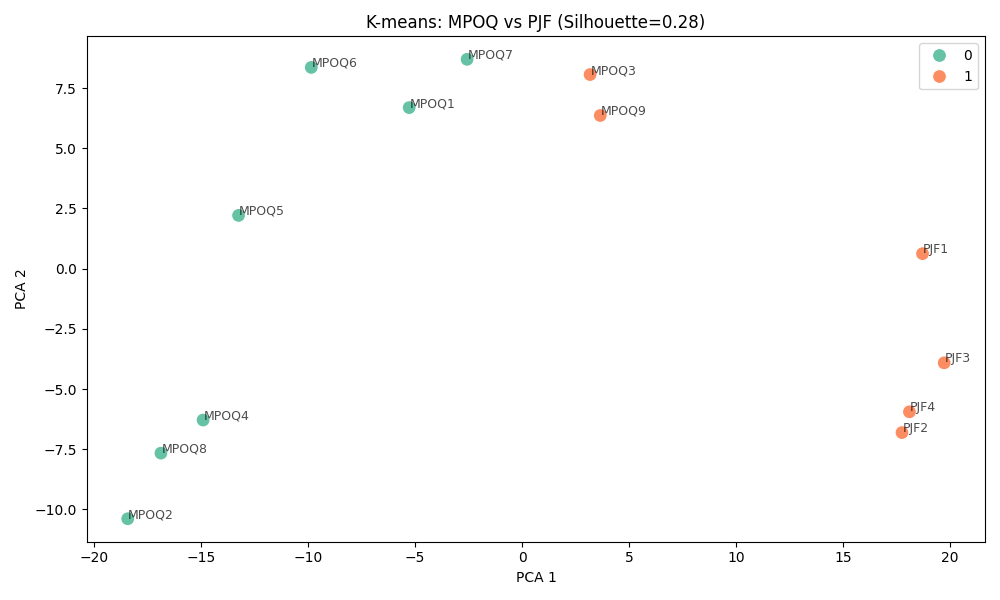


Figure 6. POQ vs P- J Fit


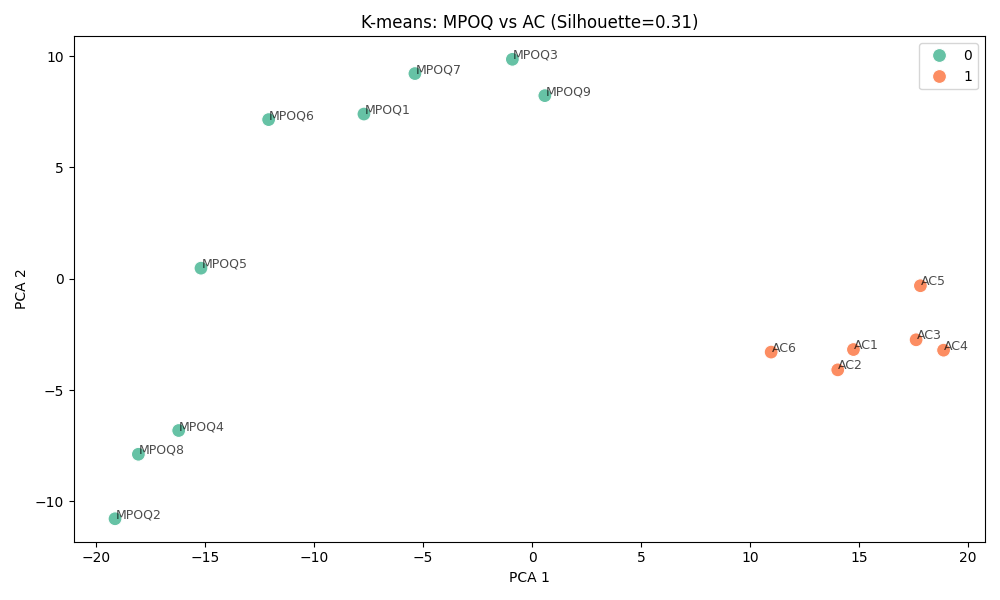


Figure 7. POQ vs Affective Commitment


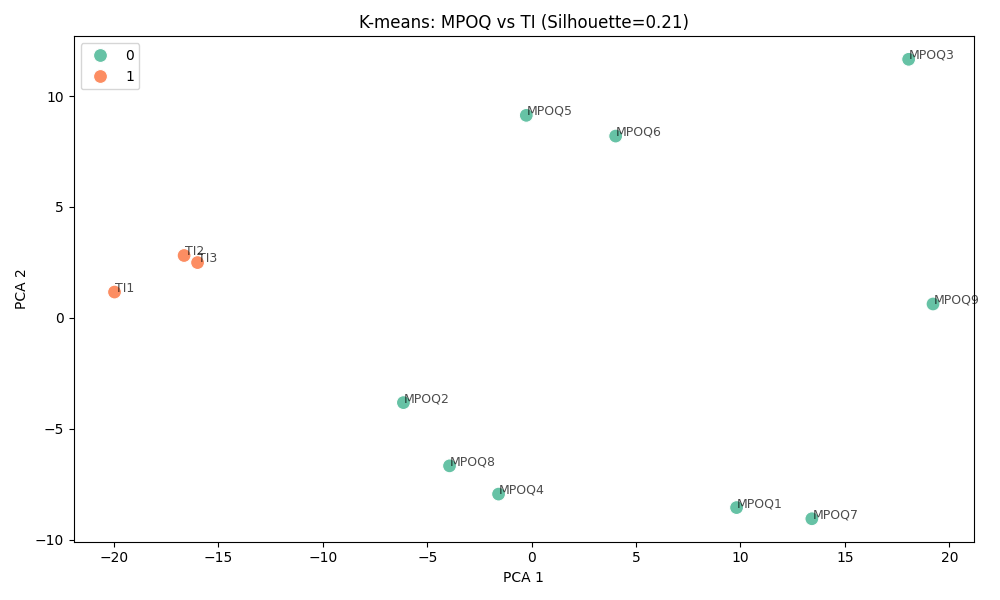


Figure 8. POQ vs Turnover Intention


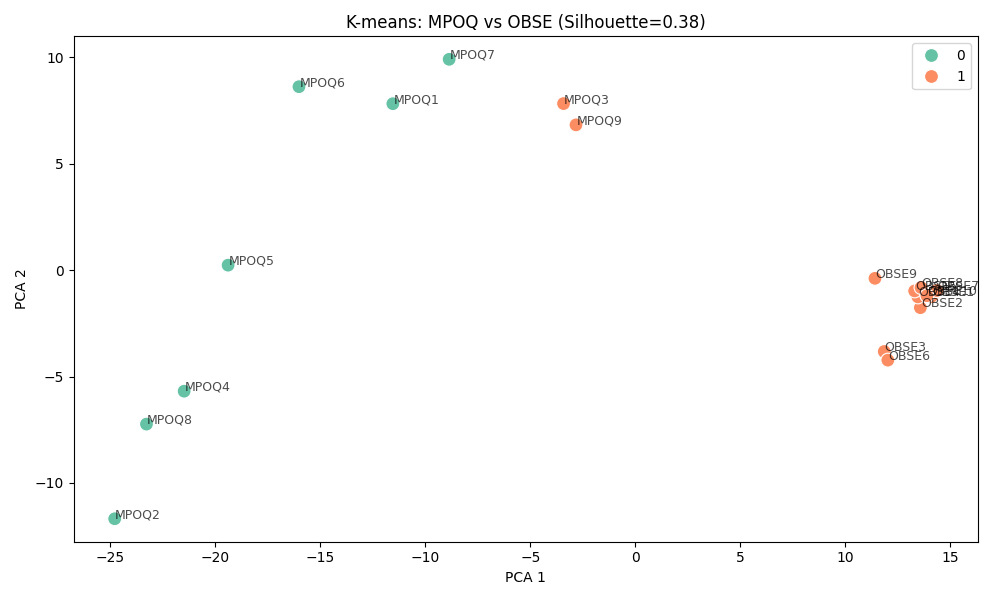

Figure 9. POQ vs Organization-based Self-esteem


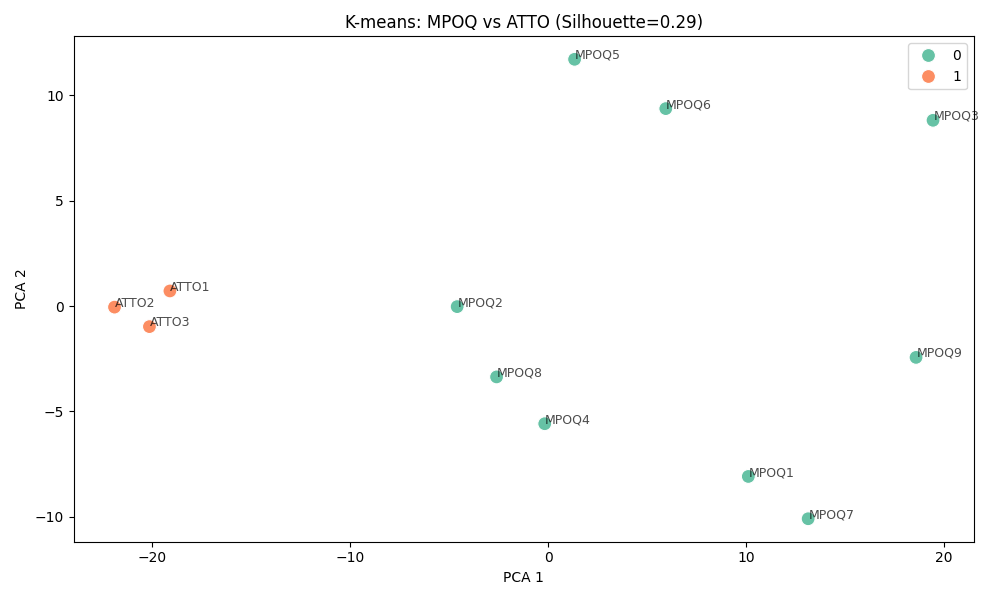


Figure 10. POQ vs Anger Toward the Organization


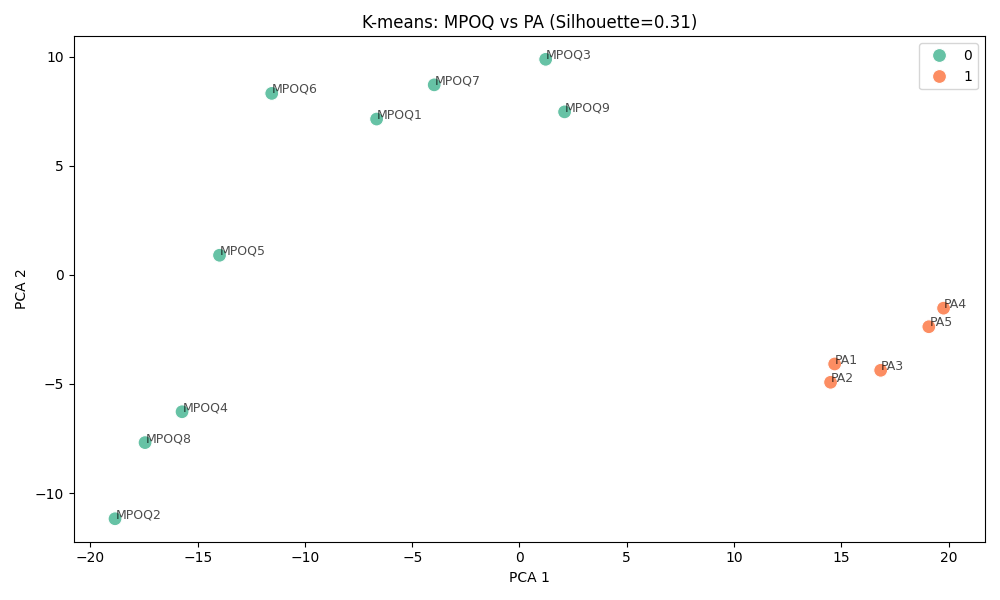


Figure 11. POQ vs Positive Affectivity


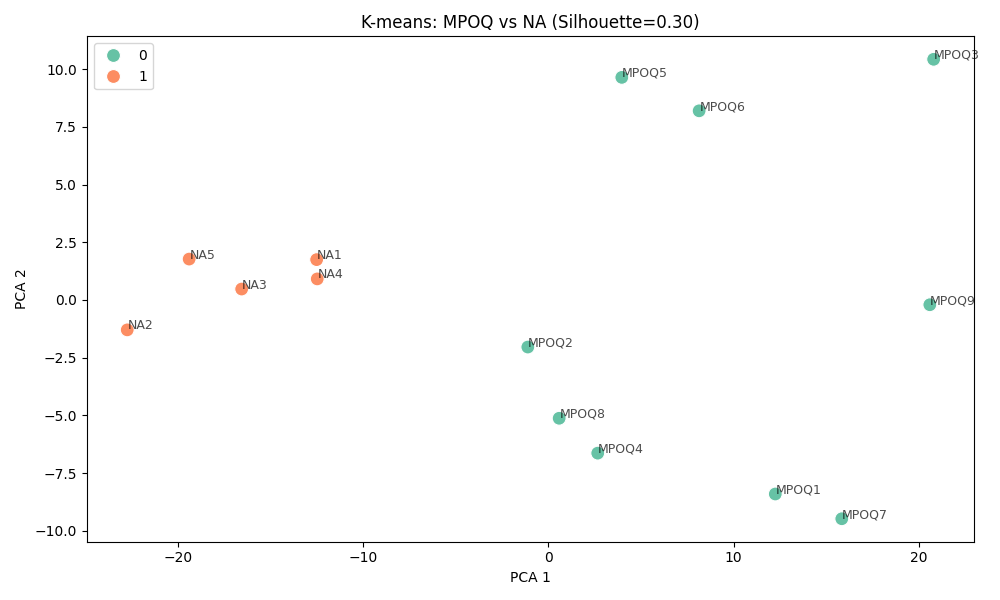


Figure 12. POQ vs Negative Affectivity
